# Liver Transplantation (LT) for Cryptogenic Cirrhosis (CC) and Nonalcoholic Steatohepatitis (NASH) Cirrhosis: Data from the Scientific Registry of Transplant Recipients (SRTR): 1994 to 2016

**DOI:** 10.1097/MD.0000000000011518

**Published:** 2018-08-03

**Authors:** Pegah Golabi, Haley Bush, Maria Stepanova, Cameron T. Locklear, Ira M. Jacobson, Alita Mishra, Gregory Trimble, Madeline Erario, Chapy Venkatesan, Issah Younossi, Zachary Goodman, Zobair M. Younossi

**Affiliations:** a Betty and Guy Beatty Center for Integrated Research, Inova Health System; b Department of Medicine, Center for Liver Diseases, Inova Fairfax Hospital, Falls Church, VA; c Department of Medicine, Mount Sinai Beth Israel Hospital, New York, NY; d Center for Outcomes Research in Liver Diseases, Washington, DC.

**Keywords:** cryptogenic cirrhosis, diabetes, MELD score, mortality, NASH, outcome, transplant registry

## Abstract

Nonalcoholic steatohepatitis (NASH)-related cirrhosis and cryptogenic cirrhosis (CC) have become leading indications for liver transplantation (LT) in the US. Our aim was to compare the trends, clinical presentation, and outcomes for transplant candidates with NASH and CC.

The Scientific Registry of Transplant Recipients (1994–2016) was used to select adult LT candidates and recipients with primary diagnoses of NASH and CC without hepatocellular carcinoma.

Two lakh twenty-three thousand three hundred ninety-one LT candidates were listed between 1994 and 2016. Of these, 16,214 (7.3%) were listed for CC and 11,598 (5.2%) for NASH. Before 2004, NASH was seldom coded for an indication for LT, but became more common after 2009. Averaged across the study period, CC candidates compared with NASH candidates were younger and had fewer conditions of metabolic syndrome (MS). CC patients were more likely to have MS components in comparison to candidates with other chronic liver diseases (CLDs) (all *P* < .0001). For most of the study period, patients with CC or NASH were similarly more likely to be taken off the list due to deterioration or death, with to patients with other CLDs. Post-LT data were available for 14,052 transplant recipients with NASH or CC. With the exception of post-transplant diabetes, the outcomes of patients transplanted for CC and NASH were similar to those of other CLD patients.

Number of LT due to CC and NASH cirrhosis is increasing. In the past decade, there is a shift from LT listing diagnosis from CC to NASH potentially related to increased awareness about NASH in transplant centers in the US.

## Abstract 2

Supplemental Digital Content is available in the text

## 1 Introduction

Despite hepatitis C virus (HCV) being the leading indication for liver transplantation (LT) in the United States (US),^[1–4]^ recent reports have indicated that listing trends for LT may be changing. In fact, nonalcoholic steatohepatitis (NASH) as an indication for LT has risen substantially so that it is currently the second most common indication for LT in the US,^[5,6]^ synchronously with a steady increase in the prevalence of nonalcoholic fatty liver disease (NAFLD) and its complications in the US.^[1–4]^ This trend is also affected by a simultaneous decrease in the number of patients with HCV requiring LT because of highly effective and safe antiviral treatments for HCV infection,^[7–9]^ while no similarly effective treatment option exists for NAFLD. In fact, it is predicted that the combination of these 2 factors could push NASH or the progressive form of NAFLD, to become the most common indication for LT in the near future.^[10–12]^

It is important to remember that, until relatively recently, most of the clinical research related to NASH combined the diagnostic category of NASH-related cirrhosis and cryptogenic cirrhosis (CC).^[13,14]^ In fact, over a decade ago, evidence suggested that the majority of patients with CC in the US may have had “burnt out” NASH.^[5,13,15–20]^ Nevertheless, controversy remains if patients transplanted for CC have NASH with the same on-list and post-LT outcomes or whether the 2 diagnoses indeed can be used interchangeably for most of the affected patient population. Therefore, the objective of this analysis is to use longitudinal data from the national registry to compare the trends and outcomes of patients listed or transplanted for NASH or CC in the US.

## 2 Methods

### 2.1 Data source

In this study, we used data from the Scientific Registry of Transplant Recipients (SRTR), which includes data on all donor, wait-listed candidates, and transplant recipients in the US, submitted by the members of the Organ Procurement and Transplantation Network (OPTN). The Health Resources and Services Administration (HRSA), U.S. Department of Health and Human Services provides oversight to the activities of the OPTN and SRTR contractors.

For this study, we included all liver transplant candidates and recipients of at least 18 years of age who were waitlisted or transplanted in 1994 through 2016 with the primary diagnosis of NASH or cryptogenic or idiopathic cirrhosis (the NASH+CC cohort). Patients with hepatocellular carcinoma (HCC) and acute liver failure were excluded from that cohort. Patients with all other causes of chronic liver disease (CLD; without HCC or indications of acute liver failure) who had been waitlisted or transplanted in the same years were used as non-NASH non-CC controls.

For all candidates, the studied outcomes were transplantation and wait list dropout; the latter included death or deterioration while waiting. For transplant recipients, the outcomes were being discharged alive after transplantation versus inpatient death before discharge, being obese (BMI ≥30) and having type 2 diabetes in follow-up, as well as postdischarge mortality (determined by matching with the Social Security Death Master File provided by SRTR), and graft loss (defined by either documented retransplant, or by a cause of death that indicated graft failure). Patients undergoing retransplants were included in the mortality analysis only with their most recent transplants. Patients with no documented date of death were presumed alive as of March 1, 2017. Candidates who were presumed alive and had no documented wait list removal indication were presumed to be still waitlisted.

### 2.2 Statistical analysis

All collected clinical and demographic parameters of included waitlisted candidates and transplant recipients were summarized as mean ± standard deviation or N (%) and were compared between patients with NASH, CC, and with other CLD using Chi-square or Mann–Whitney test without and with accounting for the year of listing/transplantation. For assessment of time trends, Kendall correlation coefficients were calculated. *P* values of .05 or less were considered significant.

All analyses were run in SAS 9.4 (SAS Institute, Cary, NC). The study was granted a nonhuman subject research status by Inova Institutional Review Board.

## 3 Results

### 3.1 Clinicodemographic characteristics of candidates with NASH and cryptogenic cirrhosis

**Figure 1:**
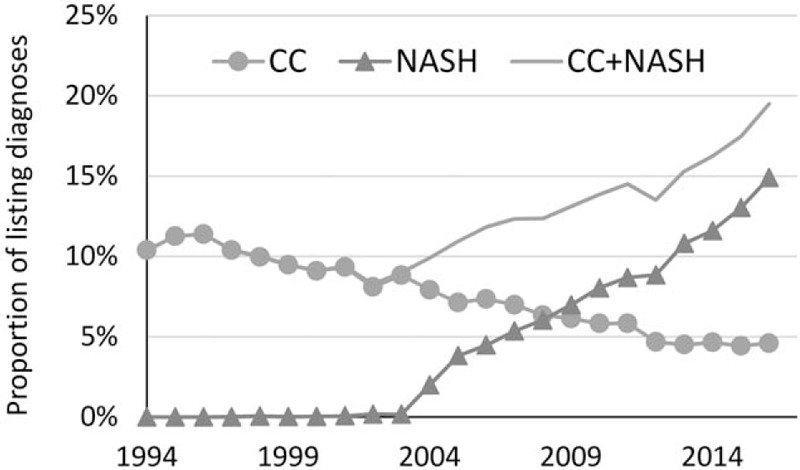
The prevalence of cryptogenic cirrhosis (CC) and NASH over time among candidates listed for liver transplantation, by year of listing.

Over the study period (1994–2016), a total of 223,391 adult LT candidates were listed. Of these, 16,214 (7.3%) were listed for CC and 11,598 (5.2%) for NASH without HCC, with both accounting for >12.5% of all candidates were listed for LT. From 1994 to 2016, there have been partially mutually exclusive trends in the rates of CC and NASH listings among transplant candidates (Fig. 1). Indeed, the diagnosis of NASH was not used until 2004, and then it grew substantially (by approximately 1.1 percentage points per year) so that it became more prevalent than CC by 2009. In contrast, the rate of CC was decreasing throughout the study period. Despite this, the total CC+NASH rate also increased over time, from the lowest 8.3% in 2002 to the highest 19.5% in 2016 (Fig. 1).

Averaged across the study period, patients with NASH were older than those with CC (58.9 vs 55.7 years), but both were still older than patients listed for LT with other CLDs (51.9 years) (all *P* < .001). Candidates with CC were also more likely to be male than patients with NASH (52.7% vs 50.3%). White race comprised (82.3%) of NASH candidates and only (75.3%) of CC candidates (*P* < .001). The model for end-stage liver disease (MELD) scores were highest among NASH candidates (22.4) followed by candidates with CC (21.1), and the rates of cirrhosis complications and some comorbidities (stroke, dialysis, cancer) were also higher in NASH (Table 1).

**Table 1:**
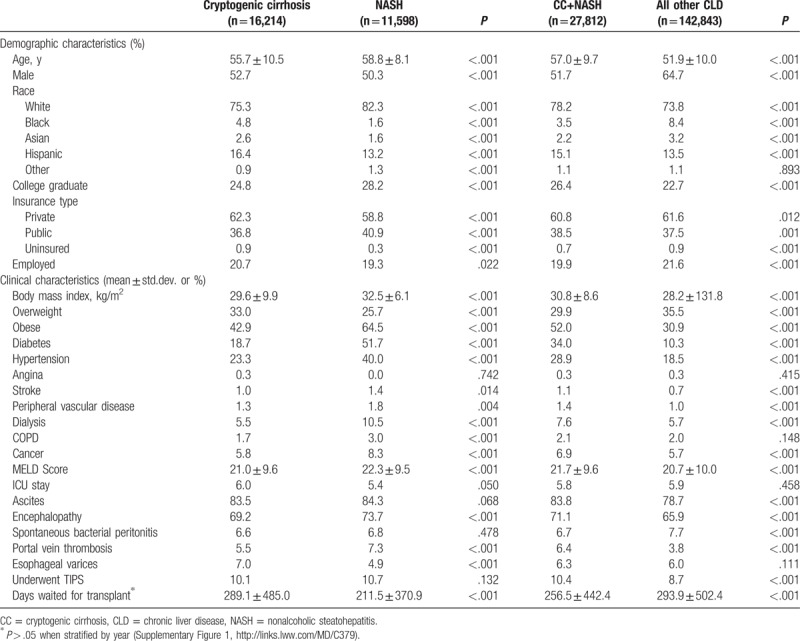
Demographic and clinical characteristics of liver transplant candidates (% or mean ± std.dev.).

### 3.2 Metabolic syndrome components in patients listed for liver transplantation

**Figure 2:**
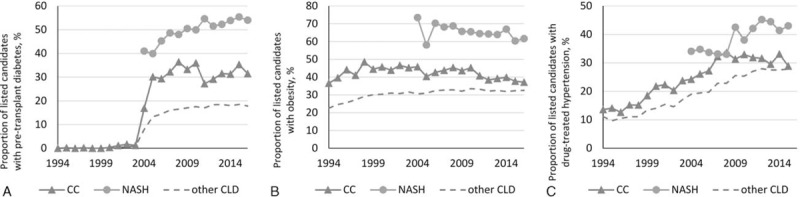
Prevalence of (A) diabetes, (B) obesity, and (C) hypertension among cryptogenic cirrhosis (CC) and NASH candidates over the study period. All *P* < .0001.

Between the study years, there were similar trends in the metabolic profile of patients with CC and NASH (Fig. 2). In fact, before 2004, there were almost no pretransplant diabetes recorded in any LT candidates. On the contrary, starting in 2004, the prevalence of diabetes in NASH exceeded 40% and continued to grow to approximately 55% in 2010s. The rate of pretransplant diabetes in CC was a bit lower, ranging between 30% and 35% in the same years, although it was still substantially higher than in other CLD controls (14–18%) (all *P* < .0001) (Fig. 2A).

A similar trend in pretransplant obesity and a less pronounced but correlated trend in pretransplant hypertension were also observed (Fig. 2B, C).

### 3.3 Wait list outcomes for patients with NASH versus cryptogenic cirrhosis

**Figure 3:**
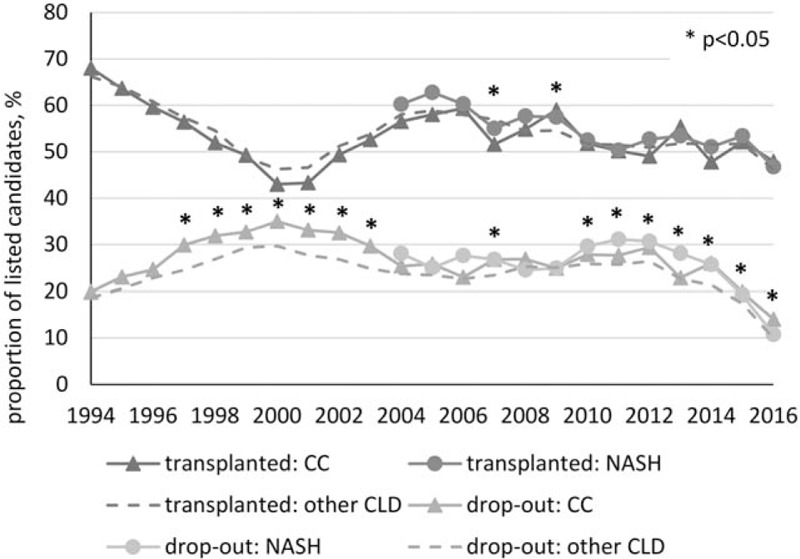
Outcomes of liver transplant candidates with cryptogenic cirrhosis and NASH.

Over the study period, patients listed with the diagnosis of CC or NASH had similar rates of receiving LT (average 53.3% vs 53.1%, all *P* > .10). When all CC and NASH candidates were compared with candidates listed with all other CLDs (average transplant rate 53.7%), there was also no significant difference (Fig. 3). The average wait time until transplantation was similar in CC, NASH, and other CLD candidates when adjusted for the year of listing (Supplementary Figure 1; *P* < .05 for 2014–2016 only). On the contrary, the average dropout rate from LT list for candidates with CC (27.3%) was significantly higher than candidates with NASH (24.0%); this, however, was completely explained out by the difference in timing (*P* > .025 in each given year). Despite this, the dropout rate in NASH and CC was consistently higher in comparison to patients with other CLDs (average 23.9%, *P* < .05 for years 1997–2003, 2007, and 2010–2016) (Fig. 3).

### 3.4 Post-transplant outcomes of patients transplanted for NASH versus cryptogenic cirrhosis

**Figure 4:**
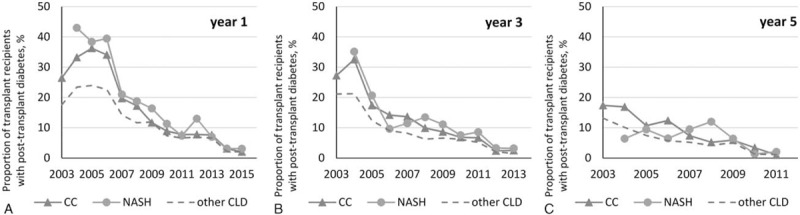
Post-transplant diabetes in transplant recipients with CC and NASH (all but one *P* > .05 between CC and NASH).

**Figure 5:**
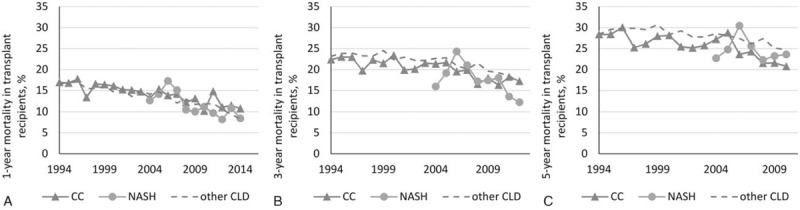
Post-transplant mortality in transplant recipients with CC and NASH (all *P* > .04 between CC and NASH).

Transplant data were available for 8137 CC, 5915 NASH, and 79,153 other CLD recipients (which is 94–96% of candidates removed from the wait list due to receiving a transplant). Clinicodemographic data were similar to that reported above for listed candidates. Post-LT prevalence of diabetes was similar in NASH and CC at all time points, and was higher than in other CLD until 2012 when the rates of post-transplant diabetes decreased substantially in all cohorts (to less than 8% by year 1, less than 4% by year 3) (Fig. 4). In addition, there was no difference in post-transplant cancer (2.2% in CC, 2.5% in NASH, 2.1% in other CLD by year 3, *P* > .20), mortality (Fig. 5), or graft loss rates when adjusted for the year of transplant (all *P* > .05).

## 4 Discussion

This study compares the trends in the prevalence, clinical characteristics, and outcomes of candidates who were listed or transplanted with the diagnosis of NASH and CC in the United States. Our data show a clear increase in the number of cases who are listed for LT in the US with the combined diagnoses of NASH and CC. In addition, there is a clear shift in the listing diagnosis from CC to NASH. This is most likely due to better recognition of NASH as a diagnostic category for listing for LT and the increasing number of cases with NASH-related cirrhosis seen in the US. Interestingly, in comparison to CC, NASH subjects who were listed or transplanted were older and more likely to be white and female. This is consistent with the demographic profile of NAFLD and NASH patients from the general population.^[21–25]^

An important finding of our study is that, in comparison to patients with CC, patients with NASH have a higher prevalence of components of metabolic syndrome, other comorbidities, and higher MELD scores. Again, this is consistent with a number of other studies showing that NASH subjects who are listed for LT have more obvious evidence for metabolic abnormalities than those listed with the diagnosis of CC.^[24–32]^ On the contrary, it is important to note that NASH patients were primarily listed in more recent years, so that increasing relaxation of contraindications for LT plays a role, in consistence with the increasing trends in the prevalence rates of comorbidities in all diagnostic groups.^[33]^ Furthermore, the rates of metabolic syndrome components were still substantially higher in CC in comparison to other CLD patients. On the basis of the pretransplant diabetes rates, we believe that a large proportion of CC patients listed for LT have underlying NASH. The exact proportion of CC patients with burned-out NASH cannot be determined but can be estimated between 50% and 75%.^[19,34,35]^ This estimate is based on pretransplant diabetes and other metabolic abnormalities, which are very susceptible to the development of severe liver disease.

In comparison to other CLDs, a similar proportion of patients with CC and NASH eventually received LT, and the dropout rates were also similar in NASH versus CC, although those rates were higher than in other CLD, primarily due to lower proportion of NASH+CC patients being removed from the list because of improvement. In contrast to this finding, a report by O’Leary et al^[23]^ suggested that NASH/CC had a lower chance of receiving LT than patients with HCV.

Post-transplant outcomes in patients with CC, NASH, and other CLD were largely similar with the exception of post-transplant diabetes, which was similarly higher in CC and NASH in comparison to other CLD patients. These data suggest that CC and NASH patients have similar potential for recurrence of metabolic abnormality and probably share the same pathogenic pathway that led to their cirrhosis. It is important to note that more recently, post-LT diabetes has decreased regardless of the diagnostic group. This could probably be explained by the use of less diabetogenic immunosuppressants in the recent years.

Our data also showed that post-transplant mortality and graft failure was not different between CC and NASH, after adjustment for the year of transplant. These findings are not consistent with a study by Singal et al^[36]^ who reported that both graft and patient survival rates at 1, 3, 5, and 10 years after LT were worse for patients with CC than NASH, even though NASH patients were older, and had metabolically worse picture than CC before LT. It is important to note, however, that that study did not account for patients with NASH being listed and transplanted primarily in more recent study years when all post-transplant outcomes improved solely due to recent advances in the transplant procedure and post-transplant management.

Although this study has many strengths, our main limitation was the availability of the initial data in the SRTR database, which has many missing records across fields, especially in earlier study years. In particular, pretransplant diabetes was not recorded until 2004, which does not allow for more robust estimates of metabolic profile of patients with CC.

In conclusion, NASH as an indication for LT has become increasingly more recognized in the past decade. This may be due to both increasing prevalence of NASH and increasing recognition that most CC patients do, in fact, have NASH. Nevertheless, CC patients without components of metabolic syndrome before LT may have other etiologies rather than pure NASH. Despite this possibility, LT candidates with CC and NASH have similar on-list and post-LT outcomes. Further prospective studies are needed to determine why and how some patients with NASH lose hepatic steatosis, as they develop more advanced cirrhosis labeled as CC.

## Author contributions

PG, HB, IY, and CTL participated in the study design, helped the interpretation of the data, and drafted the manuscript. MS performed the statistical analysis and helped the interpretation of the data. AM, GT, ME, CV, and ZG participated in the interpretation of the data and revised the manuscript. ZG, IMJ, and ZMY conceived the study, participated substantially in its design and coordination, and helped to draft the manuscript. All authors read and approved the final manuscript.

**Conceptualization:** Alita Mishra, Gregory Trimble, Madeline Erario, Zobair M. Younossi.

**Data curation:** Maria Stepanova, Zachary Goodman.

**Formal analysis:** Maria Stepanova.

**Investigation:** Pegah Golabi, Haley Bush, Chapy Venkatesan, Issah Younossi.

**Methodology:** Maria Stepanova.

**Resources:** Issah Younossi, Zachary Goodman.

**Supervision:** Ira M. Jacobson, Zobair M. Younossi.

**Validation:** Ira M. Jacobson, Chapy Venkatesan, Zachary Goodman, Zobair M. Younossi.

**Writing – original draft:** Pegah Golabi, Haley Bush, Maria Stepanova, Cameron T. Locklear, Alita Mishra, Chapy Venkatesan, Issah Younossi.

**Writing – review & editing:** Ira M. Jacobson, Gregory Trimble, Madeline Erario, Zachary Goodman, Zobair M. Younossi.

Author name: Orcid number:

Zobair M. Younossi: 0000-0001-9313-577X

Pegah Golabi: 0000-0001-9818-0983

## Supplementary Material

Supplemental Digital Content
