# Supplementary material for: Liver Transplantation (LT) for Cryptogenic Cirrhosis (CC) and Nonalcoholic Steatohepatitis (NASH) Cirrhosis: Data from the Scientific Registry of Transplant Recipients (SRTR): 1994 to 2016
Source: Medicine (Baltimore). 2018 Aug 3;97(31):e11518. doi: 10.1097/MD.0000000000011518 (PMC6081090; doi:10.1097/MD.0000000000011518)

**Supplementary Figure 1**. Median waiting time till transplant in CC and NASH candidates by year. All p>0.05.


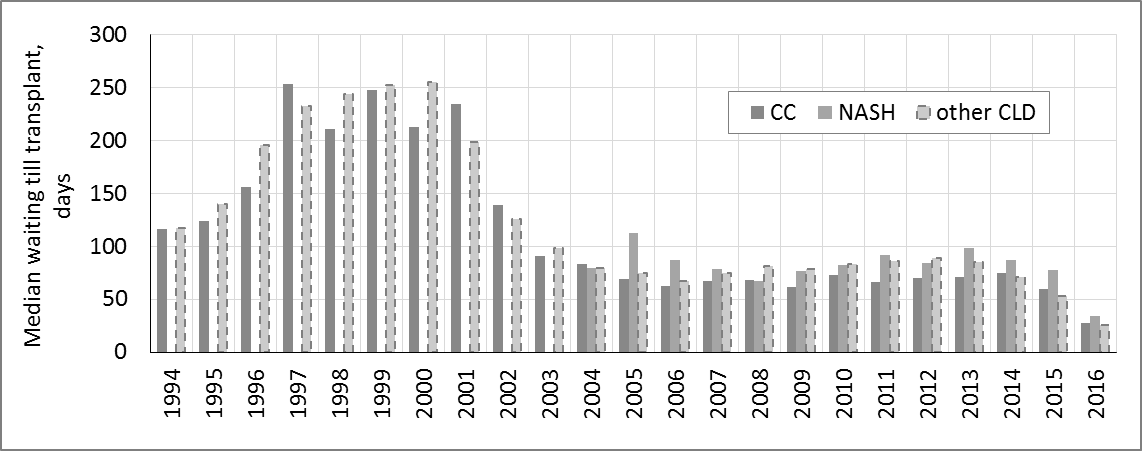

Supplement: Supplemental Digital Content [file medi-97-e11518-s001.doc]
